# Dielectric Properties of Nanoconfined Water from *Ab Initio* Thermopotentiostat Molecular Dynamics

**DOI:** 10.1021/acs.jctc.2c00959

**Published:** 2023-01-27

**Authors:** Florian Deißenbeck, Stefan Wippermann

**Affiliations:** † Max-Planck-Institut für Eisenforschung GmbH, Max-Planck-Straße 1, 40237 Düsseldorf, Germany; ‡ Philipps-Universität Marburg, Renthof 5, 35032 Marburg, Germany

## Abstract

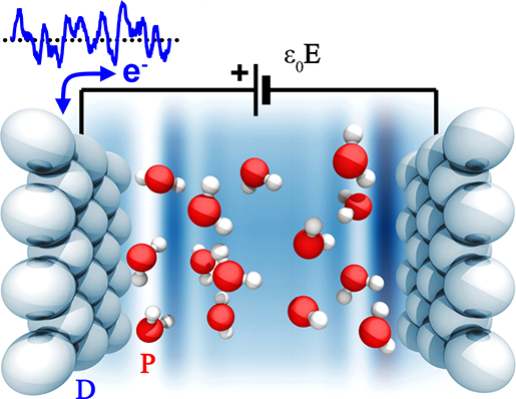

We discuss how to include our recently proposed thermopotentiostat technique [Deissenbeck et al. Phys. Rev. Lett.2021, 126, 13680333861101] into any existing *ab initio* molecular dynamics (AIMD) package. Using thermopotentiostat AIMD simulations in the canonical NVTΦ ensemble at a constant electrode potential, we compute the polarization bound charge and dielectric response of interfacial water from first principles.

Electrochemical processes occurring at the interface between a solid electrode and an aqueous electrolyte are central to future sustainable energy conversion and storage solutions.^1,2^ Applying a voltage across these interfaces in order to achieve control over the reaction pathways and kinetics is the defining concept of electrochemistry.^3^ At electrified interfaces, however, water forms stratified layers^4^ with properties that differ strongly from those observed in bulk solutions.^5^ Therefore, solvent reorganization in response to the electric field, ion de/resolvation processes, the formation of the electric double layer, and charge transfer reactions all proceed within these interfacial water regions with modified properties.

In order to reveal the precise mechanistic details of these processes, it is critical to develop accurate simulation techniques to explore and predict structural properties and chemical reactions at electrified surfaces in contact with liquid electrolytes from first principles. While experiments are routinely performed at a constant electrode potential, realizing these conditions in *ab initio* molecular dynamics (AIMD) simulations has remained very challenging. A suitable AIMD potentiostat technique requires two constituents: (i) a robust method to either apply an electric field or charge the electrode and (ii) an algorithm to control either the field or charge in accordance with the thermodynamic theory in order to drive the system to the desired electrode potential.

Multiple solutions have been suggested for issue (i). The modern theory of polarization (MTP)^6,7^ explicitly includes the field inside the simulation cell, that is generated by moving the corresponding charge from one boundary of the unit cell to the opposite one. The charge itself is outside the unit cell. This approach has been used to perform first-principles calculations with either a constant total electric field **E**(7) or a constant electric displacement **D**(8) as electrostatic boundary conditions. Alternatively, Lozovoi and Alavi proposed to perform constant potential calculations by including an explicit compensating counter charge inside the unit cell to ensure charge neutrality.^9^ More recent approaches build on these ideas to either apply directly an electric field^10−15^ or include explicit compensating counter charges.^17−23^

We note that without exception the techniques outlined above rely on electrostatic boundary conditions that enforce that either the total electric field **E** or the electric displacement **D** is kept exactly constant during the AIMD run. In the thermodynamic sense, all these methods describe purely microcanonical ensembles with constant total energy. In the thermodynamic limit, averaged properties are independent of the chosen ensemble. If, however, the observables of interest explicitly depend on fluctuations (e.g., reaction mechanisms and rates, etc.) or if the simulated system is small, the microcanonical ensemble may not be used. Instead, the electrode charge must be treated as a thermodynamic degree of freedom, allowing it to react to the dynamics of the solvent and to charge transfer processes. Such a treatment requires canonical sampling (issue (ii)).

The first such approach was pioneered by Bonnet et al.^24^ and later extended by Bouzid et al.^25,26^ Bonnet et al. suggested to describe the electrode charge by second order dynamics coupled to a Nosé–Hoover thermostat. This approach, however, requires “*[*...*] the existence of an energy function**that is differentiable with respect to the total electronic charge. This implies the ability to treat noninteger numbers of electrons and, in general, non-neutral systems.*”^24^ Unfortunately, in the context of density-functional calculations, the total energy as a function of the number of electrons is a notoriously difficult quantity to compute. Furthermore, the electronic charge is a single degree of freedom. Yet, controlling single degrees of freedom by the Nosé–Hoover method often leads to non-ergodic behavior. In order to recover ergodicity, the introduction of Nosé–Hoover chains was proposed,^27^ however, at the cost of additional numerical parameters, and required extra tuning. In order to lift these requirements and enable a straightforward implementation of the potentiostating process into any simulation package, we were recently inspired by the MTP^6^ and the Maxwell-Langevin equations of fluctuation electrodynamics^28^ to introduce a stochastic canonical thermopotentiostat algorithm.^29^

Here, we discuss the implementation of our thermopotentiostat technique in the context of electronic structure calculations and *ab initio* molecular dynamics. Specifically, we choose to build our implementation on the computational counter electrode (CCE) recently proposed by Surendralal et al.^21^ In contrast to the finite field methods described in refs (6−20, 22, and 23), which are available in only some of the most commonly used density-functional theory (DFT) codes, the CCE technique has the added advantage that its application does not require any changes inside the electronic structure code. However, we emphasize that the thermopotentiostat algorithm is equally straightforward to implement using any of the methods outlined in refs (6−20, 22, and 23).

Figure 1 illustrates the computational setup chosen in the present study. The simulation cell contains an electrolyte or dielectric medium (explicit or implicit) that is enclosed between a working and a reference electrode, carrying equal and opposite charges *n* and – *n*, respectively. Hence, the simulation cell is charge-neutral in total. The working electrode is connected to an external reservoir of charge at constant electron chemical potential, so that the *external* voltage difference Φ_0_ between the working and reference electrodes is exactly constant. The potential Φ_0_ is the independent thermodynamic variable that can be controlled in experiments. The *system* potential Φ inside the simulation cell, in contrast, is defined as the difference of the workfunctions on the vacuum side and the solvated surface of the working electrode, cf. Figure 1. It is neither a constant nor necessarily equal to the bath potential, due to the microscopic size of the region targeted by our simulations and the exchange of charge with the external environment. Instead, the system potential Φ as well as the Fermi level depend on the evolution of the system, as the charge *n* reacts to the dynamics of the solvent.

**Figure 1:**
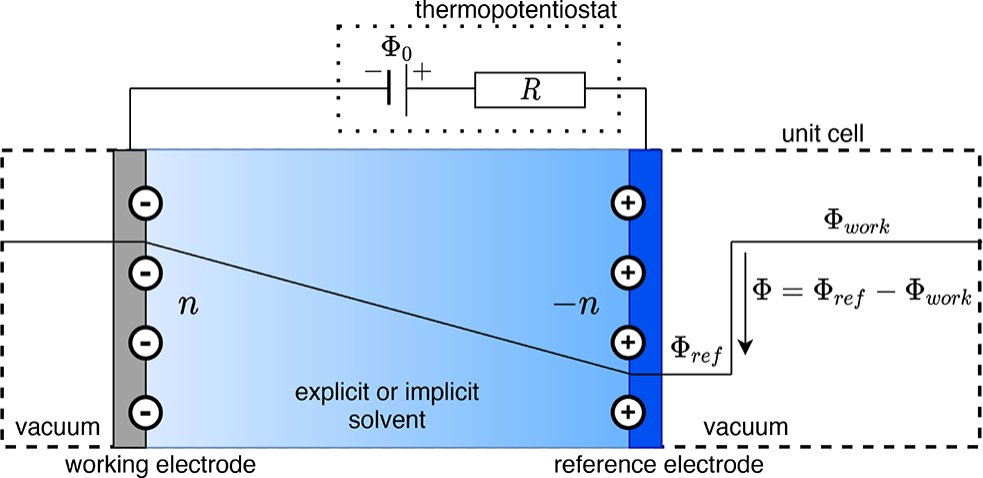
Schematic representation of the computational setup. The periodic simulation cell is indicated by the dashed line. The power supply and resistor located outside the unit cell represent the thermopotentiostat introduced in ref (29). As an input quantity, the thermopotentiostat requires the instantaneous potential Φ. It is determined from the difference of the workfunctions on the vacuum side and solvent covered surface of the working electrode, and it is equal to the total dipole moment of the charges contained in the simulation cell along the surface normal. The potential as drawn corresponds to the electron potential, consistent with the definition commonly used in electronic structure codes. By convention, we choose that an increasingly positive potential corresponds to an increasingly positive charge on the working electrode.

Controlling the charge *n* at each discrete simulation time step allows us, in principle, to drive the system potential Φ toward the desired target value for the external bath potential Φ_0_. Treating thereby the system potential as a thermodynamic degree of freedom implies, however, that the system is able to perform external work and, hence, dissipate energy. In order to uphold energy conservation, the energy loss due to controlling the system potential must be balanced exactly, on average, by a corresponding energy gain from thermally induced fluctuations. The physical system realizes this condition by coupling to a fluctuating electric field created by temperature dependent charge fluctuations due to the thermal motion of the electrons and ions.^30^ To mimic this behavior, a potentiostat must apply an electric field with an explicit finite temperature and hence become a “thermopotentiostat”. We introduced such an algorithm in ref (29) and derived a direct expression for the electrode charge *n* at each discrete time step
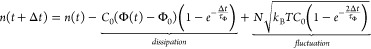
1where *n* is the electrode charge, and *N* is a random number drawn from a Gaussian distribution with zero mean and variance one. *C*_0_ is the geometric capacitance of the bare electrodes in the absence of the dielectric or solvent, and τ_Φ_ ≔ *RC*_0_ is the potentiostat relaxation time constant. The instantaneous system potential Φ(*t*) of the working electrode with respect to the reference electrode is obtained from the total dipole moment of the charges contained in the simulation cell parallel to the surface normal.^29^ In practice, Φ(*t*) is computed using the dipole correction scheme^31^ that is available in most density-functional codes. We use the convention that an increase of the potential in the positive direction implies an increasingly anodic polarization on the working electrode, indicated by the vertical arrow in Figure 1.

We note that eq 1 determines at each discrete time step only the total amount of charge *n* located on the finite segment of the working electrode enclosed within our periodic simulation cell. In the context of Born–Oppenheimer (BO) DFT, the actual distribution of *n* on the electrode surface is determined by the electronic energy minimization. However, since in the BO approximation there is no explicit electron dynamics and hence no scattering, the electrode charge is redistributed instantaneously at each time step, effectively describing an electrode with infinite surface conductivity. Therefore, the physical meaning of the resistance *R* shown in Figure 1 – and by extension the relaxation time τ_Φ_ in eq 1 – is to introduce an effective mean surface conductance that governs the flow of charge into and out of the finite segment of the electrode described within the simulation cell.

In practice, τ_Φ_ is set to a sufficiently small value to enable an efficient sampling of the phase-space but not yet small enough to disturb the system dynamics. Note that the mean and the variance of the charge as given by eq 1 are unaffected by the choice of τ_Φ_. Small values of τ_Φ_, however, correspond to a large damping factor and may thus adversely affect the dynamics of the system if set too aggressively. In general, a time-constant longer than the slowest vibrational frequency present in the system is a reasonable choice. We therefore adopt τ_Φ_ = 100 fs as a default value.

Moreover, note that the present first-principles computational setup differs from the semiempirical one described in ref (29) in another important aspect: in BO-DFT calculations, the simulated system is instantaneously polarizable. Since the capacitance enters eq 1 as an adjustable parameter, the potentiostating process seemingly requires prior knowledge about the dielectric properties of the system. This, however, is not the case. In fact, eq 1 was designed to take the instantaneous polarizability correctly into account. This property of eq 1 can be understood intuitively, considering that the instantaneous electric current  is independent of the capacitance. It is only the discrete change *n*(*t* + *Δt*) – *n*(*t*) that depends on the capacitance. For convenience, the construction of eq 1 treats any deviations in the actual capacitance *C* = ϵ_*r*_*C*_0_ with respect to the parameter *C*_0_ within the time domain: even if ϵ_*r*_ contains a contribution due to instantaneous polarizability, eq 1 is guaranteed to sample the correct statistical distribution for the charge *n* with σ_*n*_^2^ = *k*_B_*Tϵ*_*r*_*C*_0_, albeit with an increased relaxation time of ϵ_*r*_τ_Φ_. An analytical proof of this important but counterintuitive property inherent to eq 1 is included in the SI.^32^

Conceptually, the parameter *C*_0_ plays a role analogous to the mixing parameter β of the charge mixing schemes^33−36^ commonly used in DFT, where β determines how much of the old density is mixed to the new density from one electronic iteration to the next. In case one of the dimensions of the unit cell is significantly larger than the other two, small changes of the electron density with respect to the more extended direction are associated with large changes in the total energy. In essence, such a unit cell corresponds to a reduced capacitance *C*_0_, which scales with 1/*l*, where *l* is the length of the unit cell. Thus, in order to prevent charge sloshing and convergence issues, it is often necessary to reduce the mixing parameter β in these situations. The parameter *C*_0_, like β, is of purely numerical nature and has no impact on any physical observable. However, it must be chosen appropriately to ensure numerical stability. The exact value of *C*_0_ is uncritical, and its choice is straightforward: setting *C*_0_ to approximately ϵ_0_*A*/*d*, where *A* is the area of the unit cell parallel to the electrode surface, and *d* is the distance between the working and the reference electrodes that resulted in stable convergence behavior in all cases investigated.

We now turn to discuss the implementation of our thermopotentiostat into existing AIMD packages. The implementation must be built on top of a method to realize either a finite charge on the working electrode or apply a finite electric field. The thermopotentiostat is completely general and can be used to control either the field or charge in conjunction with any of the methods described in refs (6−23). As a basis for our implementation, here we chose the computational counter electrode (CCE) recently proposed by Surendralal et al.,^21^ because the CCE can be directly used with any DFT code. Building on the CCE, only the thermopotentiostat needs to be implemented inside the electronic structure code as a control scheme in analogy to a thermostat but not the finite field method itself.

In their scheme, Surendralal et al.^21^ used a large band gap insulator, so that the Fermi level of the total system can be controlled within the electronic gap of the CCE by doping. To transfer charge between the working electrode and the CCE, Surendralal et al. suggested to dope the CCE using pseudoatoms with fractional core charges *Z*_*CCE*_. This approach places an adjustable charge on the working electrode and at the same time provides an equal and opposite compensating counter charge on the CCE.

In the following, we couple the thermopotentiostat to the CCE: at each ionic step, the thermopotentiostat is used to determine the change of the charge *n* that is located on the segment of the working electrode described within the simulation cell, exchanged with an external bath at constant electron chemical potential. The new electrode charge is then realized by adjusting the core charge of the atoms constituting the CCE according to
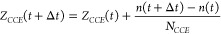
2where *N*_*CCE*_ is the number of atoms constituting the counter electrode, and *n* is computed according to eq 1.

Most BO-DFT codes rely either on the (velocity) Verlet or leapfrog algorithms to integrate the equations of motion. Figure 2 outlines a code structure commonly used throughout many existing DFT packages, complemented by our thermopotentiostat. After the calculation of the electronic structure and the forces at time *t*, both the positions and the electrode charge (or *Z*_*CCE*_, respectively) can be updated directly and in any order to time *t* + *Δt*. The code structure differs slightly if velocity Verlet integration is used, cf. Figure S1(32) for a representative example.

**Figure 2:**
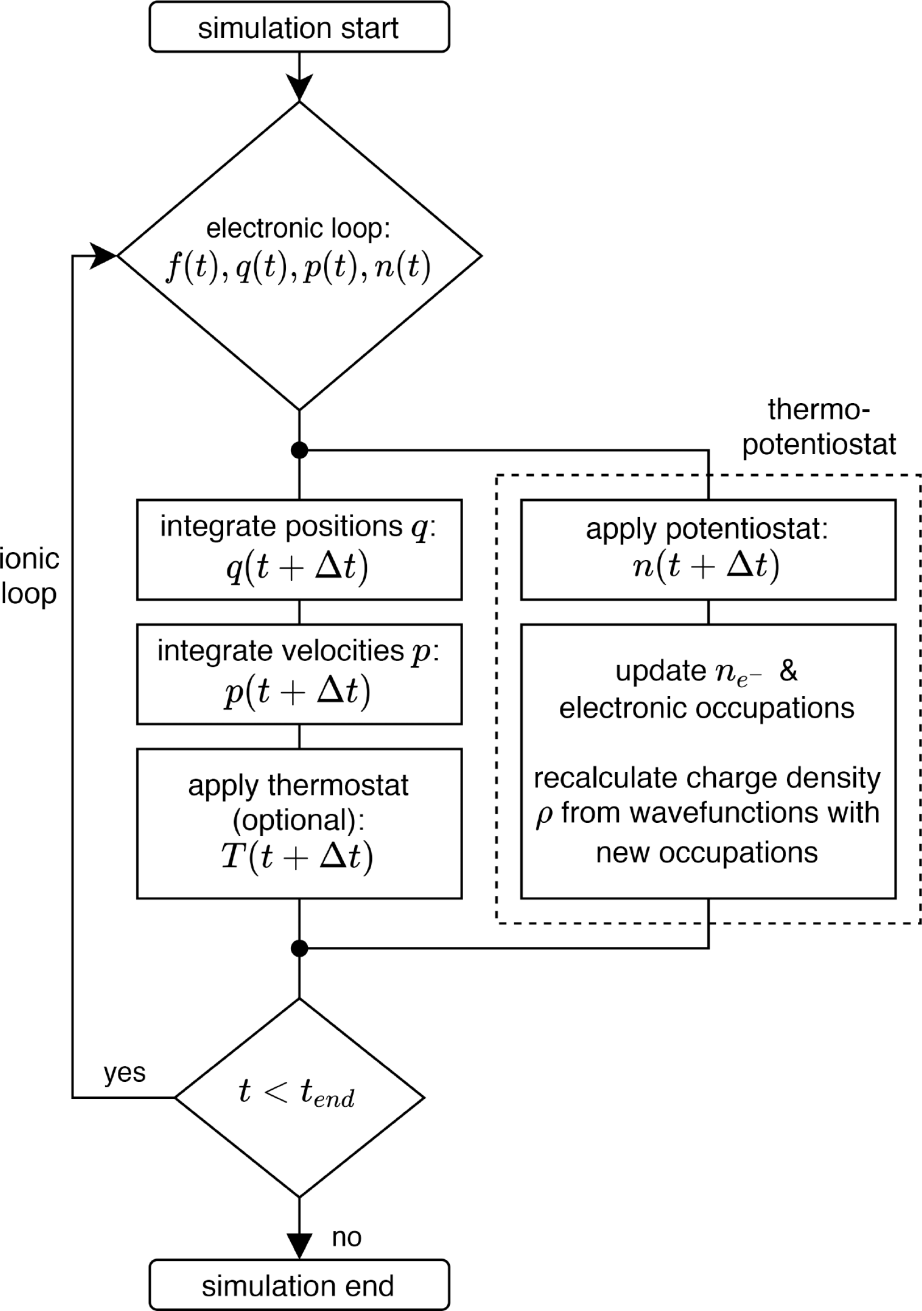
Flowchart of the thermopotentiostat implementation in conjunction with leapfrog integration of the equations of motion, see text. A corresponding flowchart for velocity Verlet integration is included in the SI.^32^

We note that although our computational setup guarantees that the total system is always charge neutral, the number of electrons *n*_*e*^–^_ contained inside the simulation cell is free to change from one ionic step to the next, as the thermopotentiostat adjusts the compensating counter charge located on the CCE. After computing the updated electrode charge *n*(*t* + *Δt*) and doping the CCE according to eq 2, it is therefore necessary to update also the number of electrons *n*_*e*^–^_ perceived by the electronic structure code. If the number of electrons changes, by default, most DFT codes will shift the electron density by a constant offset so that its volume integral becomes equal to the new electron number. A straightforward shift of the electron density, however, may cause charge sloshing during the electronic minimization and led to convergence issues in previous approaches.^9^ The presence of explicit fluctuations in our approach exacerbates this problem further.

To ensure that the electronic loop converges reliably, we note that in the physical system charge is added to or removed from the electrode at the Fermi level only. In order to recover the physically correct behavior and prevent charge sloshing in our setup, we recalculate the electronic occupations for the electronic structure at time *t* but with the already updated number of electrons *n*_*e*^–^_(*t* + *Δt*). Subsequently, the electron density ρ is recalculated from the present wave functions at time *t* using the new occupations and introduced into the Hamiltonian at time *t* + *Δt*. We tested this approach at the example of different semiconducting and metallic working electrodes, and we obtained a completely robust electronic convergence behavior in all cases investigated.

In order to highlight the opportunities provided by our *ab initio* thermopotentiostat technique, we now turn to a topic that currently attracts considerable attention: interfacial water and water under confinement feature structural and dynamic properties that differ significantly from those of bulk water.^4,5,37^ Most notably, thin water films confined to a few nm thickness exhibit a strongly reduced dielectric response in the direction perpendicular to the confining surfaces.^5^ Since the high polarizability of liquid water is regarded as the origin of its unique solvation behavior and interfacial water is omnipresent, it is necessary to understand the mechanism and to be able to accurately simulate the dielectric response of liquid water at realistic interfaces.

The work by Fumagalli et al.^5^ therefore stimulated a considerable number of theoretical studies, cf. e.g. refs (38−43). Most studies used the Kirkwood-Fröhlich theory^44−46^ or the theory of polarization fluctuations^47^ in order to compute the dielectric tensor from the variance of the total dipole moment fluctuations per volume. Converging these variances enforces, however, a statistical sampling of the water dynamics spanning a time scale of several hundred ns. For this reason, atomistic simulations of interfacial water’s dielectric response have been largely restricted to force-field approaches and nonpolarizable water models.

Moreover, experiments, such as ref (5), measure static dielectric constants averaged over multiple molecular layers. Hence, there has been continued interest to use the computed local dielectric profiles^38−40^ to calculate static dielectric constants that can be directly compared to experiments. Such a calculation requires the dielectric volume as an input parameter. In the context of force-field molecular dynamics, ref (40) suggested to define the volume via the dielectric dividing surface positions. In the presence of adsorbates, charge transfer, and thermal motion of the electrode surface, for curved/corrugated interfaces or in the context of explicit electronic structure simulations as pursued here, however, the exact location of the boundary for an interface between an electrode and a dielectric may be harder to define.

The need for such a definition reflects an assumption implicit to the construction of both the Kirkwood-Fröhlich theory and the theory of polarization fluctuations: these approaches describe the dielectric response of the system enclosed within the periodic simulation cell to a displacement field created by sheet charges that are placed exactly on the boundaries of the simulation cell. In *ab initio* simulations of electrified interfaces, however, the distribution of the electrode charge may differ significantly from idealized sheet charges, a situation which the Kirkwood-Fröhlich theory and the theory of polarization fluctuations are fundamentally unable to account for.

Both problems are solved by our thermopotentiostat technique in conjunction with explicit applied fields. Introducing the densities of the electrode charge *n* and the bound charge *n*_*p*_ as the explicit quantities to describe the response of a dielectric medium exposed to an electric field enables direct and parameter-free access to dielectric properties. Moreover, the densities can be computed at least 2 orders of magnitude faster than dipole variances, due to the use of thermodynamic averages and the efficient stochastic canonical sampling of our approach. Themopotentiostat MD thereby opens the door toward simulations of interfacial dielectric properties from first principles.

We implemented our thermopotentiostat approach into the Vienna Ab Initio Simulation Package (VASP) and performed AIMD simulations for liquid water confined between two computational Ne electrodes, using the generalized gradient approximation PBE.^48,49^ As test cases we considered ensembles consisting of 32, 64, and 192 water molecules, corresponding to electrode separations of *d* = 10.7, 17.4, and 31.4 Å, respectively. Consistent with the work of Fumagalli et al.,^5^ we applied potentials of Φ_0_ = 0 and 4 V, respectively. Further numerical details are provided in the SI.^32^

In Figures 3a and b, we show the time evolution of the potential difference and the charge transferred between the two electrodes, respectively, directly after switching on the thermopotentiostat. The system potential is driven efficiently toward the externally applied voltage and becomes stationary after a simulation time of ∼4 ps has elapsed. Yet, a net current continues to flow until ∼*t* = 8 ps, where the charge assumes an equilibrium value of *n̅* = −1.73 *e*^–^, cf. Figure 3b. This electric displacement current is caused by the increasing polarization density of the water due to the reorientation of the water molecules within the applied electric field. This reorientation occurs on a much slower time scale than the changes in the applied electric field, which are governed by the chosen relaxation time constant of τ_Φ_ = 100 fs. Moreover, after the system has reached equilibrium, the electrode charge continues to fluctuate (inset Figure 3a). We note that two different processes contribute to these fluctuations: on the one hand, any water dynamics that are associated with a change in the total dipole moment, as well as contributions due to electronic screening, are actively countered by the thermopotentiostat in order to drive the potential toward its target value. This action of the thermopotentiostat therefore takes the form of deterministic fluctuations in the electrode charge. On the other hand, the energy dissipated due to potential control is returned, on average, by the stochastic fluctuations introduced in eq 1.

**Figure 3:**
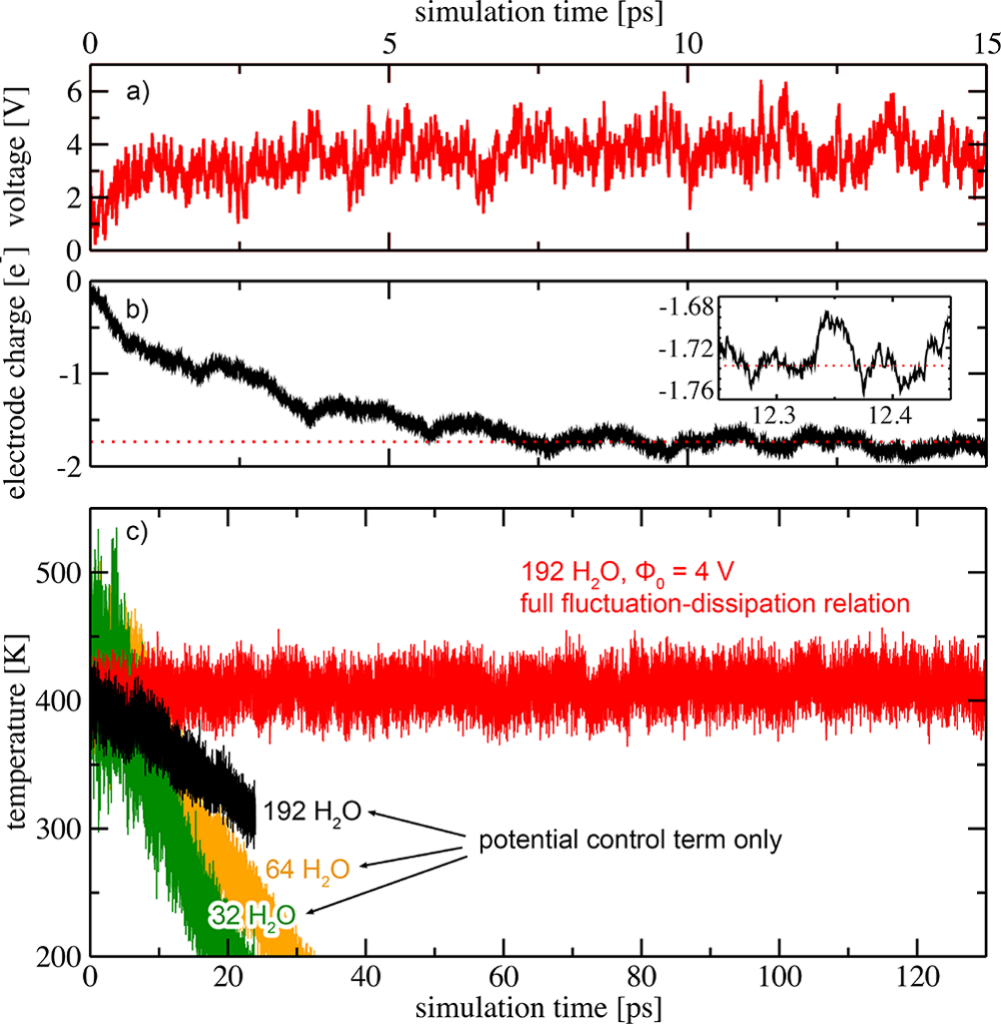
**a)** Time evolution of the electrode charge *n* and **b)** the system potential Φ for an NVE ensemble consisting of 192 H_2_O molecules, potentiostated to Φ_0_ = 4 V. The red dotted line marks the average electrode charge after equilibration. The inset shows the stochastic charge fluctuations in an enlarged region around the average electrode charge. **c)** Time evolution of the temperature for multiple NVE ensembles consisting of 32, 64, and 192 H_2_O molecules, respectively, potentiostated to Φ_0_ = 4 V. The electrode charge is adjusted, using only the dissipation (potential control) term in eq 1 (green, orange, and black lines) or using the full eq 1 (red line).

An important aspect of simulations under potential control is to ensure that the interplay between the deterministic potential control mechanism and the stochastic fluctuations balances out to a zero net energy change, in order to keep the system in thermodynamic equilibrium, sampling the canonical ensemble at constant temperature and applied potential. For verification, we performed all simulations presented here in the absence of an explicit thermostat. Neglecting the fluctuation term in eq 1, the potential control mechanism alone always dissipates thermal energy. For typical DFT system sizes, the pure potential control mechanism is able to drive the ensemble severely out of equilibrium in a matter of ps, cf. Figure 3c (black, orange, and green curves, respectively).

Naturally, here one may be tempted to replenish the energy dissipated due to potential control via an explicit thermostat. This is inadvisible, however, as a thermostat acts indiscriminately on all degrees of freedom, whereas a potentiostat affects only those vibrational degrees of freedom that couple to a change in the ensemble’s dipole moment parallel to the direction of the applied electric field. Draining energy from one set of degrees of freedom and subsequently returning it to another invariably leads to a spurious energy transfer between them. Such an approach cannot restore the system to equilibrium but would instead exacerbate the problem further.

If, in contrast, the full fluctuation–dissipation relation eq 1 is used (cf. Figure 3c, red curve), the thermopotentiostat eliminates any artificial net energy drain, and the temperature remained constant, on average, over the whole course of the simulation. We emphasize that these findings corroborate our claim that our central working eq 1 explicitly takes instantaneously polarizable systems into account, cf. Supporting Information.^32^

Note that by design, the fluctuation term rebalances the energy loss to potential control only when the temperature of the simulation system is equal to the target temperature specified within the fluctuation term. This property of eq 1 stems from the fact that the dissipative term implicitly depends on the system temperature, whereas the fluctuation term does not. As both terms balance out exactly only when the system and target temperatures are equal, the thermopotentiostat not only acts as a potentiostat but also will actively thermostat the system at the same time.

For simulating, e.g., reaction events, the use of an additional explicit thermostat is, fundamentally, superfluous. In practice, however, adding a dedicated thermostat may be highly desirable, e.g., for faster and more efficient equilibration. The presence of the potential fluctuations ensures that spurious energy transfers between the thermostat and thermopotentiostat, such as the one described above, are fundamentally impossible. It is now straightforward to add an explicit thermostat, as indicated in Figure 2. We expect in particular stochastic temperature control schemes to work well in conjunction with the thermopotentiostat, such as, e.g., the Lowe-Anderson thermostat,^50^ Langevin dynamics,^51^ and CSVR.^52,53^

We now explore the dielectric properties of nanoconfined water, using our thermopotentiostat technique to include the atomistic details of the interface at the AIMD level of theory. In Figure 4, we compare electrostatic potential profiles that were obtained for two different external applied voltages Φ_0_ = 0 V (blue curve) and Φ_0_ = 4 V (red curve), respectively, each time-averaged over a trajectory length of 125 ps. We partitioned the space between the electrodes into three different regions: (i) a hydrophobic gap with a thickness of 2 Å, formed between the electrode surface and interfacial water, (ii) an interfacial water region with a thickness of 7.4 Å, and (iii) a bulk-like water region, indicated in Figure 4 by areas shaded in orange, green, and gray, respectively.

**Figure 4:**
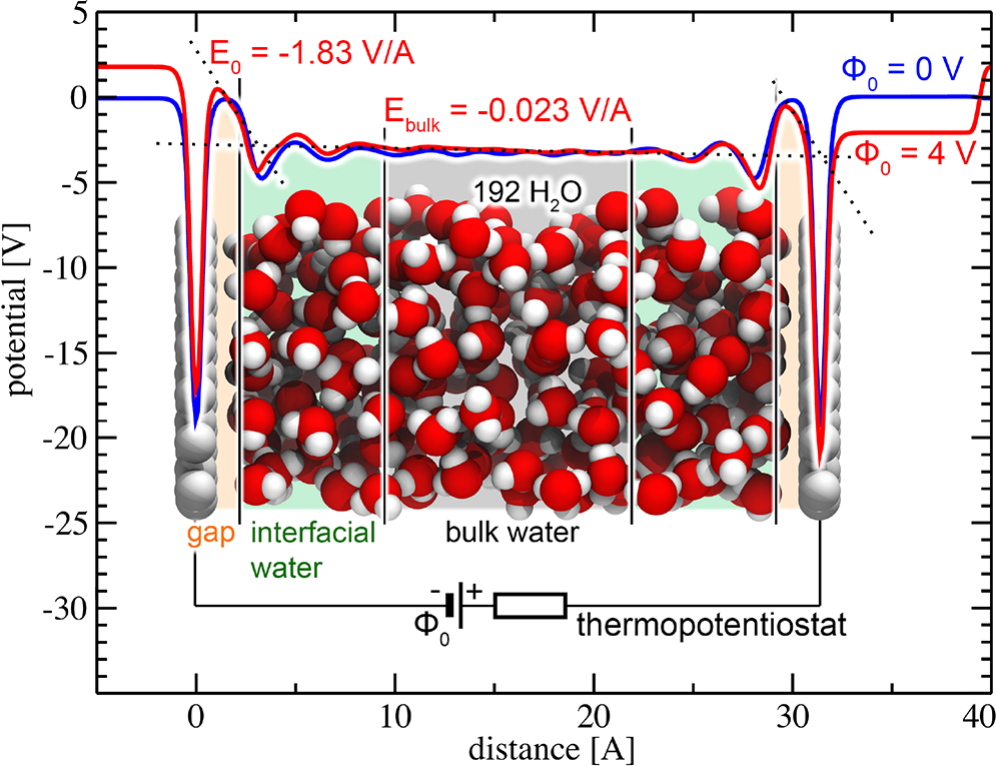
Schematic representation of the *ab initio* simulation cell. Gray balls represent electrode surface atoms (Ne), whereas red and white balls denote O and H, respectively. The unit cell has a lateral dimension of 14.5 × 14.5 Å^2^, with a distance of *d* = 31.4 Å between the electrodes, and contains 192 H_2_O molecules at the experimental density of water. Blue and red lines indicate planar-averaged electrostatic potential profiles parallel to the surface normal for applied voltages of Φ_0_ = 0 V and Φ_0_ = 4 V, respectively, time-averaged over a trajectory length of 125 ps. The beginning of the bulk-like water region is defined as the point just outside the second solvation layer of the electrode, where the potential oscillations induced by interfacial water have decayed and the number density assumes again the density of bulk water. This is the case at normal distances larger than 9.4 Å away from the positions of the electrodes.

At the positions of the electrodes, the nuclear core charges of the atoms constituting the electrodes induce deep wells in the potential. For an applied voltage of Φ_0_ = 4 V, the potential then decays linearly and unscreened within the hydrophobic gap regions, resulting in a homogeneous electric field of *E*_0,⊥_ = −1.83 V/Å determined from a separate simulation in the absence of water. The electric field is also homogeneous and constant inside the bulk-like water region. Averaging the gradient of the potential within the gray shaded region in Figure 4 yields a value of *E*_bulk,⊥_ = −0.023 V/Å. The resulting field strengths are indicated by dotted lines in Figure 4. We then estimated the static dielectric constant inside the bulk-like water region as the ratio between the unscreened electric field within the hydrophobic gap and the field in the central bulk-like region. We obtained a value of ϵ_bulk,⊥_ = *E*_0,⊥_/*E*_bulk,⊥_ ≈ 79, consistent with the one for homogeneous bulk water. [Note that the close agreement to the measured dielectric constant of bulk water is fortuitous. Since the field inside the bulk water region is only ∼1/80th compared to the vacuum case, a significantly longer sampling than our trajectory length of 125 ps is needed to determine the resulting small value of the field with sufficient accuracy in order to robustly obtain the bulk dielectric constant from the ratio of the fields.]

Although the dielectric response within the central bulk-like water region is fully consistent with the continuum picture shown in Figure 1, the region of interfacial water exhibits a distinctly different behavior. At interfaces, water forms stratified structures.^54,55^ This stratification gives rise to potential oscillations within the interfacial water region, cf. the green shaded area in Figure 4. In analogy to Friedel oscillations,^56^ which originate when screening an electric field with charge carriers of finite size, the wavelength of the potential oscillations reflects the size of the water molecules.^57^ In consequence, a considerable part of the potential drop applied between the two electrodes occurs within the hydrophobic gap, where the field is essentially unscreened, and inside the interfacial stratified water region. Since the gap and interfacial water regions are unable to effectively screen the applied electric field, the total static dielectric constant ϵ_⊥_, as measured by capacitive techniques,^5^ is significantly lower for nanoconfined water than that of homogeneous bulk water.^29^

The dielectric response of interfacial water is often characterized using spatially resolved dielectric constants and dielectric profiles.^29,38−41,58^ This approach has recently been criticized on the grounds that physically meaningful dielectric constants can only be obtained at the mesoscale, averaging over multiple molecular layers.^43^ Moreover, the ability of the interfacial water layer to polarize and store electrostatic free energy (dielectric response) should not be confused with the reduction of Coulomb interactions between charges, e.g. ions, embedded inside this region (screening). These two properties diverge at the nanoscale^42^ and cannot be described by a spatially dependent local dielectric constant, due to the granularity of the solvent.

The response of a dielectric medium to electric fields or embedded charges, however, is well-defined even at the nanoscale in terms of the induced polarization density **P** and hence–by extension–the corresponding local bound charge density ρ_bound_. We therefore propose to use ρ_bound_ as the central local quantity in the context of first-principles atomistic simulations.

In order to explicitly compute ρ_bound_, we begin by calculating the total charge density ρ_tot_ = −ϵ_0_ · *Δϕ* from the DFT effective single particle potential ϕ(**r**), according to Poisson’s equation. [We note that most *ab initio* codes define the electrostatic potential as the electron potential, which has the opposite sign compared to classical electrostatics. In Figure 4a, we adopt the same convention, so that ϕ is equal to the inverse potential shown in Figure 4a.] Note that ρ_tot_ includes both the electronic charge as well as the nuclear core charge. Subsequently, ρ_tot_ is partitioned into a free charge contribution ρ_free_, whose volume integral is equal to the electrode charge *n*, and the bound charge contribution ρ_bound_. We computed the free charge density ρ_free_ using the identical 2-electrode setup but without the water as a reference. Of course, other approaches to partition the charge density are conceivable as well, e.g. based on Wannier function techniques.^59,60^ The bound charge density is then obtained as ρ_bound_ = ρ_tot_ – ρ_free_.

Figures 5a and 5b show line profiles of the resulting bound charge densities parallel to the surface normal for applied potentials of Φ_0_ = 0 V and Φ_0_ = 4 V, respectively. For both voltages, the bound charge density is zero inside the bulk-like water region to within our numerical accuracy. This is the expected outcome. Inside the regions of interfacial water, however, the bound charge density displays characteristic oscillations, even for zero applied voltage (cf. Figure 5a). These oscillations correspond to the specific structure assumed by water at the interface, in particular the stratification discussed above. In Figure 5a, water stratification is visible as the modulation in the water number density. The bound charge density hence gives rise to a characteristic dipole moment within the interfacial water layers. The quantitative details depend on the interfacial chemistry between water and the specific electrode and may include charge transfer due to e.g. chemisorbed water.

**Figure 5:**
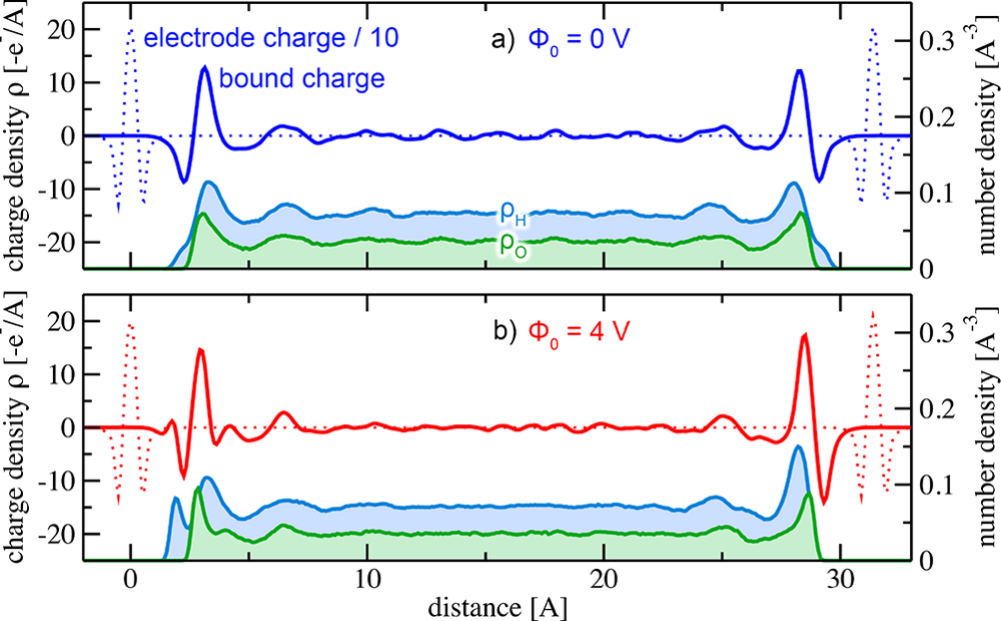
**a)** Bound charge density profiles for Φ_0_ = 0 V and **b)** Φ_0_ = 4 V, obtained by subtracting the total charge density of the electrodes as a reference, see text. For improved visibility, the electrode charge densities shown as dotted lines have been scaled by a factor of 1/10. Filled light green and light blue areas indicate O/H number density profiles, respectively.

This purely *chemical contribution* to the bound charge density is to be clearly distinguished from *field-induced* contributions due to the presence of surface charges. For an applied voltage of Φ_0_ = 4 V, Figure 5b shows distinct modifications to the bound charge density. These modifications coincide with field-induced changes to the internal structure of the solid–water interface, as illustrated by the water number density shown in the bottom of Figure 5b. Since the left electrode is negatively charged, on average, one of the hydrogen atoms of each interfacial water molecule is now pointing toward the electrode, while the other one remains available to contribute to the hydrogen bond network. This reorientation is visible in the form of a double peak structure in the H number density close to the left-hand interface. It is also reflected in the distribution of the angle between the OH-bond and the surface normal. A recent study^54^ reported similar findings for water-gold interfaces. We include the calculated angle distributions in the SI.^32^

In principle, both the *chemical* and *dielectric responses* of a solvent to a given solute can be accurately described in terms of the bound charge densities discussed above. Bound charge densities computed from first-principles, hence, represent important benchmarking quantities that allow us to evaluate the performance of implicit solvent models, such as e.g. modified Poisson–Boltzmann (MPB), models based on the integral theory of liquids (e.g., RISM), and molecular DFT.^61^

Despite the above-mentioned shortcomings^42,43^ of describing the nanoscale dielectric response using local dielectric constants, this approach is often desirable for practical reasons in the construction of implicit solvent models. There has hence been continued interest to compute local dielectric profiles^4,29,38−41,58^ for water–solid interfaces.

Such dielectric profiles can be directly computed from the bound charge densities. As a first step, we compute the polarization density *P*(*z*) = ∫_0_^*z*^ ρ_*bound*_(*z*′) d*z*′, cf. Figure 6a. For Φ = 0 V, the chemical contribution to the bound charge density due to water stratification is reflected in the nonzero polarization density close to the interfaces, but *P*(*z*) naturally vanishes inside the bulk-like water region. There is no net polarization in this case. The polarization density obtained for an applied voltage of Φ = 4 V, in contrast, clearly displays a constant net polarization. We remind the reader that the bound charge densities, and thus by extension the polarization densities, consist of (i) a chemical contribution due to the presence of the interface, superimposed with (ii) an electrostatic contribution that describes the dielectric response. In order to isolate the dielectric response, we compute the polarization density difference *ΔP*, cf. Figure 6b, to cancel out any interface related structuring. In linear response theory, this polarization density difference *ΔP* is proportional to the inverse local dielectric constant ϵ_⊥_ with *ΔE* ≈ [ϵ_0_ϵ_⊥_]^−1^*ΔD*.^39^ According to the definition of the electric displacement field *ΔD*, the electric field *ΔE* is given by *ΔE* = ϵ_0_^–1^[*ΔD* – *ΔP*].

**Figure 6:**
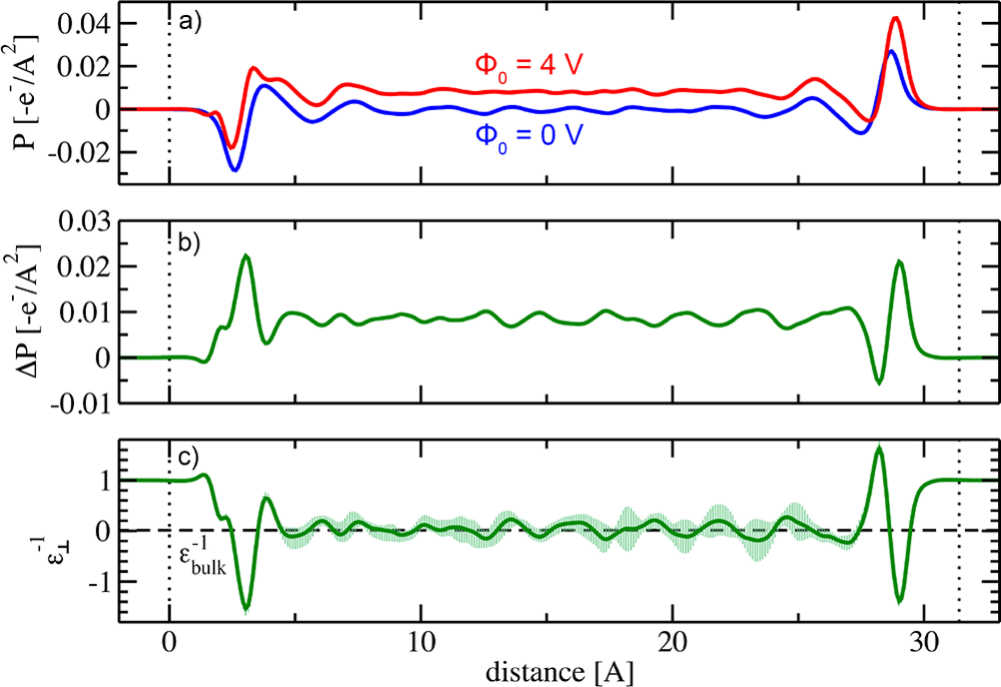
**a)** Polarization densities obtained for Φ_0_ = 0 V and Φ_0_ = 4 V, respectively, and **b)** polarization density difference. **c)** Inverse dielectric profile computed from the polarization density difference shown in **b)** with error bars. The dashed line marks the value of ϵ_*bulk*_^–1^ for liquid water. Dotted lines denote the positions of the electrodes.

Figure 6c shows the inverse dielectric profile. Compared to dielectric profiles obtained from empirical force-field approaches, which often involve statistical sampling of several nanoseconds, the present simulations were sampled for 125 ps and thus exhibit somewhat larger errors bars, indicated by vertical lines in Figure 6c. The largest error bars by far are encountered inside the bulk-like water region. Close to the interfaces, in contrast, statistical fluctuations are much less pronounced, and the interfacial dielectric features shown in Figure 6c are robust within the numerical accuracy of our simulations.

In comparison to dielectric profiles computed using force-field approaches, cf. e.g. ref (29), the first-principles dielectric profile presented here features distinct differences between the dielectric responses to the negative and positive charges located on the left- and right-hand side electrodes, respectively. Moreover, interfacial water exhibits additional structuring in the dielectric response that is not captured by empirical simulations. Most notably, the inverse dielectric constant assumes values of ϵ_⊥_^–1^ > 1 directly at the interfaces. The induced polarization of the water therefore locally enhances the applied electric field. This phenomenon is known as antiscreening and was recently proposed to occur within the electrochemical double layer.^62^ Our results demonstrate that antiscreening is already present in neat water.

In conclusion, we extended our thermopotentiostat approach toward *ab initio* molecular dynamics (AIMD) simulations and demonstrated its implementation in the context of density-functional theory. We emphasize that the thermopotentiostat can be implemented using any of the currently available finite electric field or charge techniques. In order to highlight the performance of our thermopotentiostat AIMD technique, we computed the dielectric properties of nanoconfined water from first principles. These developments allowed us to directly obtain bound charges and polarization densities due to the dielectric response of interfacial water at a constant electrode potential. Both the bound charge and the induced polarization density represent important benchmark quantities for future implicit solvent models that are able to accurately describe solvation at the nanoscale. Moreover, our thermopotentiostat AIMD technique is able to describe bond making and breaking, as well as charge transfer processes at electrified solid–liquid interfaces at a constant electrode potential. Our developments thus open the door toward simulations of electrochemical and electrocatalytic processes from first principles.

## Supporting Information Available

Additional mathematical background on how thermopotentiostat algorithm treats instantaneously polarizeable systems, numerical details of simulations, analysis of statistical errors, and discussion of orientational structure of interfacial water (PDF)
