# Supplementary material for: Dielectric Properties of Nanoconfined Water from Ab Initio Thermopotentiostat Molecular Dynamics
Source: J Chem Theory Comput. 2023 Jan 27;19(3):1035–43. doi: 10.1021/acs.jctc.2c00959 (PMC9933428; doi:10.1021/acs.jctc.2c00959)
Supplement: Supplementary file 1 — ct2c00959_si_001.pdf [file ct2c00959_si_001.pdf]

Supporting Information: Dielectric properties of nanoconfined water from *ab initio* thermopotentiostat molecular dynamics

Florian Deißenbeck† and Stefan Wippermann*,†,‡

†*Max-Planck-Institut für Eisenforschung GmbH, Max-Planck-Straße 1, 40237 Düsseldorf, Germany*

‡*Philipps-Universität Marburg, Renthof 5, 35032 Marburg, Germany*

E-mail: stefan.wippermann@physik.uni-marburg.de

Instantaneously Polarizeable Systems

Our central working equation (1) in the main paper is equally valid for non-polarizable and instantaneously polarizable systems. As derived in Ref.[S1], the fluctuation-dissipation theorem (FDT) takes the following form for the electrode charge:

$$f dt = \underbrace{-\frac{1}{\tau_\Phi}(\Phi - \Phi_0)\, dt}_{dissipation} + \underbrace{\sqrt{\frac{2}{\tau_\Phi}\frac{k_B T}{C_0}}\, dW_t}_{fluctuation}, \tag{S1}$$

where τ_Φ, Φ, Φ_0 and C_0 are the relaxation time constant, the instantaneous potential, the target potential and the capacitance of the bare electrodes in absence of a dielectric, respectively. We note that the differential dW_t of a Wiener process plays the same conceptual role

as dt: it represents an integration over time t, albeit with an infinitesimal stochastic time step dW_t using Itō integration[S2], cf. Ref.[S1].

If the system under investigation is instantaneously polarizable, e.g. in the context of Born-Oppenheimer dynamics, the form of Eq. S1 seemingly suggests that the capacitance C_0 would need to be corrected accordingly. Indeed it is possible, in principle, to describe instantaneously polarizable systems by adapting C_0 within the fluctuation term. However, this particular choice is inadvisable because it would require advance knowledge about the system's dielectric properties. Instead, the dielectric properties should be an outcome of the simulation, not a parameter entering it. We therefore propose to shift the issue of the unknown dielectric contributions to the capacitance into the time domain and to take into account *any* polarizability, instantaneous or not, *implicitly* within the deterministic term, as described in the following.

For simplicity, we set $\Phi_0 = 0$ without loss of generality and demonstrate the derivation of Eq. S1 explicitly for instantaneously polarizable systems. According to Ohm's Law and Kirchhoff's 2nd Law, the instantaneous current for the setup shown in Fig. 1 in the main manuscript is described by:

$$\frac{dn}{dt} = -\frac{\Phi}{R}, \tag{S2}$$

where n and R are the electrode charge and an effective resistance, respectively. We now assume that the capacitance is increased to an unknown value $C = \epsilon_r C_0$, where the factor ϵ_r describes an instantaneous dielectric response. According to the definition of the capacitance, the instantaneous voltage Φ is:

$$\Phi = \frac{n}{\epsilon_r C_0}. \tag{S3}$$

Substituting Eq. S3 into Eq. S2 and adding a corresponding fluctuation term $\tilde{n}\ dW_t$, we obtain:

$$dn = -\frac{1}{RC_0}\frac{n}{\epsilon_r}dt + \tilde{n}\ dW_t. \tag{S4}$$

In the canonical ensemble at finite temperature T, the variance σ_n^2 of the electrode charge n must satisfy the relation:

$$\sigma_n^2 = k_B TC = k_B T\epsilon_r C_0. \tag{S5}$$

Therefore, the fluctuation term $\tilde{n}$ in Eq. S4 must be constructed accordingly. Here we remind the reader that Eq. S4 formally represents a stochastic differential equation (SDE) of the so-called Ornstein-Uhlenbeck type:

$$dx = -kxdt + \sqrt{D}dW_t. \tag{S6}$$

The variance of Eq. S6 has been derived analytically using Itō calculus[S2]:

$$\sigma_x^2 = \frac{D}{2k}. \tag{S7}$$

Hence, we directly obtain an expression for $\tilde{n}$, using Eq. S7:

$$dn \;=\; \underbrace{-\frac{1}{\tau_\Phi}\frac{n}{\epsilon_r}\ dt}_{dissipation} + \underbrace{\sqrt{\frac{2}{\tau_\Phi}k_B T C_0}\ dW_t}_{fluctuation}, \tag{S8}$$

with $\tau_\Phi := RC_0$. Clearly, taking the variance of Eq. S8 according to Eq. S7 satisfies Eq. S5. Yet, we note that the fluctuation term is free of ϵ_r. By design, the deterministic dissipation term is the only place where ϵ_r needs to be introduced.

We remind the reader, that our central working equation (1) in the main manuscript is derived solving the Itō integral

$$dn = C_0\ f dt. \tag{S9}$$

Straightforward multiplication of Eq. S1 with C_0 in order to obtain dn and substituting for Φ using Eq. S3 yields:

$$dn = C_0\ f dt \ \ = \ \ -\frac{1}{\tau_\Phi} C_0 \underbrace{\frac{n}{\epsilon_r C_0}}_{=:\Phi} \ dt + \sqrt{\frac{2}{\tau_\Phi} k_B T C_0}\ dW_t, \tag{S10}$$

With Eq. S10, it is now clear that Eqs. S1 and S8 are formally identical. Our central working equation (1) therefore already takes the polarizability - instantaneous or not - implicitly into account.

Numerical and technical details

We carried out density functional theory (DFT) calculations within the Perdew-Burke-Ernzerhof generalized gradient approximation[S3], using plane wave basis sets and projector augmented wave pseudopotentials[S4] with an energy cutoff of 400 eV. All calculations were performed using the Vienna Ab Initio Simulation Package (VASP)[S5,S6]. We used the Γ point for $\mathbf{k}$-space integration. To integrate the equations of motion in our AIMD simulations and ensure accurate energy conservation over a time scale of several 100 ps, we converged the electronic total energies to 10^{-8} eV at each ionic step, used a discrete time step $\Delta t = 0.5$ fs and the 2nd order leapfrog scheme as implemented in VASP.

The simulation cells contain 2 computational Ne electrodes[S7] with a lateral size of 14.5×14.5 Å^2, separated by d = 10.7 Å, 17.4 Å and 31.4 Å, respectively, and include 32, 64 and 192 H_2O molecules between the electrodes, respectively. The values for the electrode separation d were chosen so that the bulk water density of 1 g/cm^3 is reached in the central part of the unit cell, after equilibration for 10 ps with the Langevin thermostat and a relaxation time of $\tau = 50$ fs.

From here on, the thermostat was switched off without exception and we sampled the

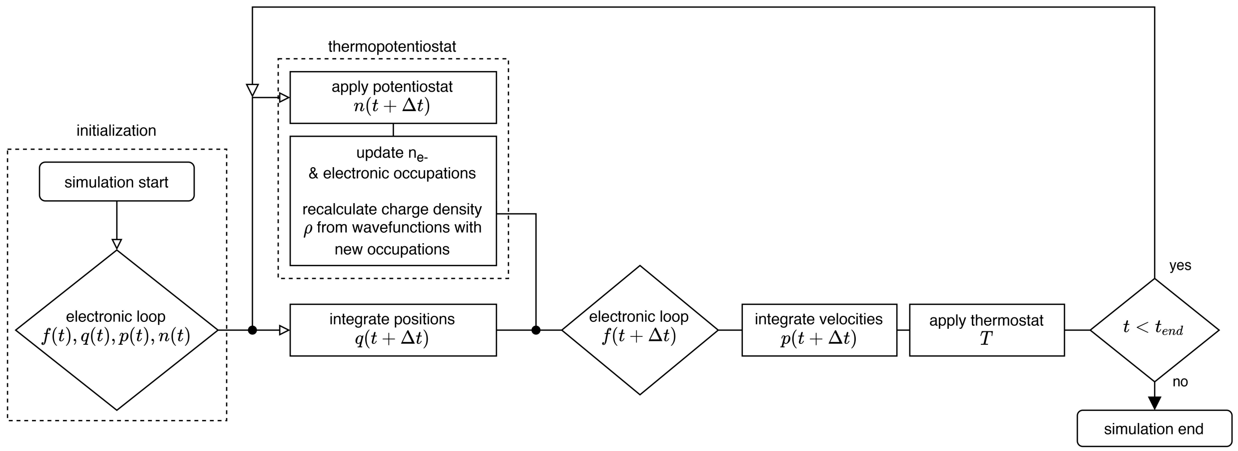

Figure S1: Flowchart to conceptually show the integration of the thermopotentiostat in second order velocity verlet. The potentiostat acts on the charge and positions of the first force calculation and updates the charge according to the thermopotentiostat or any other control logic. The new charge and the new positions are used to calculate the new forces and thereby the new velocities and the next ionic step is performed.

ensembles for an additional 125 ps. In all simulations, we integrated the equations of motion for the spatial degrees of freedom with the leapfrog scheme in the NVE ensemble. In addition to potentiostating the system, the temperature is actively controlled by our thermopotentiostat due to exposing the atoms to the fluctuating electric field, so that the simulation samples the NVTΦ ensemble.

Both computational Ne electrodes are charged by equal and opposite amounts. The amount of charge transferred between both electrodes is controlled by our thermopotentiostat. To that purpose, we use distinct Ne pseudopotentials for the left-hand side and right-hand side Ne electrodes, respectively. The core charges of the pseudopotentials describing the Ne electrodes are adjusted over the course of the simulation at each individual ionic step, according to our central working equation (1). For the thermopotentiostat relaxation time we use a value of $\tau_\Phi = 100$ fs.

Velocity Verlet integration scheme

In the main text we provided a flowchart for integration via leapfrog. Velocity Verlet is another widely used integration scheme. Here, the thermopotentiostat must be be included in a slightly different way, cf. Fig. S1. After the initialization and a first calculation of the forces the electrode charge is updated together with the positions. Subsequently, a new calculation of the forces is performed which includes the updated charge and updated positions. Next, the velocities are integrated and, if necessary, a thermostat can be applied. The integration loop is then closed by a new integration of the positions and electrode charges.

Convergence of the bound charges and dielectric profiles

Computing dielectric constants from molecular dynamics simulations is commonly performed using Kirkwood-Fröhlich theory or the theory of polarization fluctuations. Both approaches rely on the variance of the dipole moment fluctuations, typically requiring several nanoseconds of statistical sampling to obtain converged results. Our approach outlined in the main text uses only thermodynamic averages, which converge significantly faster.

In order to determine the statistical sampling necessary to converge the dielectric properties, we computed the dielectric constants within the bulk and interfacial water regions as a function of sampling time. Statistical error bars are obtained as running variances:

$$\sigma(z,t) := \mathrm{Var}\left(\frac{1}{t}\int_0^t dt' \epsilon_\perp(z,t')\right), \tag{S11}$$

where $\epsilon_\perp(z,t')$ denotes the dielectric constant at position z' at timestep t'. The position-dependent error bars of $\epsilon(z)$ for the total sampling time are shown in Fig. 6c in the main text. In Fig. S2 we show the evolution of the error bars as a function of time for the interfacial and bulk water regions. For interfacial water, the standard error of ϵ^{-1} falls below 0.1 after

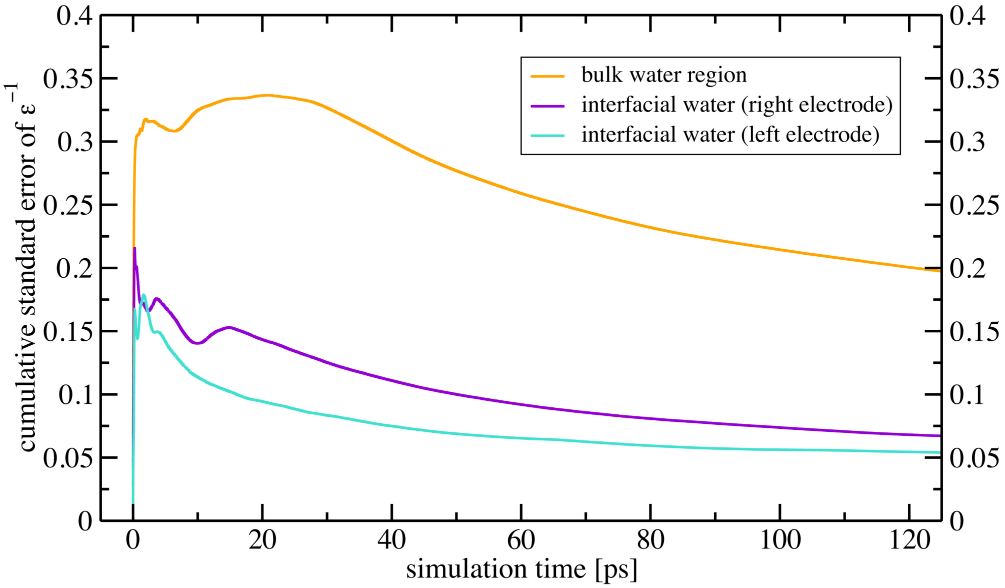

Figure S2: Evolution of the variances of the water dielectric constant in different regions with increasing statistics. This plot serves as a guideline to estimate the required number of steps to get a sufficiently accurate dielectric profile.

a sampling time of 50 ps. Consistent with Fig. 6c in the main text, the dielectric properties of interfacial water converge significantly faster compared to those of bulk water, since the water reorientation dynamics is less pronounced close to the interface.

Interfacial water structure

In order to probe the orientation of interfacial water in response to the applied electric bias, we computed the probability distributions of the angles enclosed between the surface normal and the water bisector (α, Fig. S3a) or the water OH-bond (θ, Fig. S3b). Solid and dashed lines refer to the left hand side (negatively charged) and right hand side (positively charged) electrodes. We consider only the first layer of interfacial water up to a normal distance of 4 Å with respect to the electrode, corresponding to the density minimum between the first and the second stratified water layer, cf. Fig. 5 in the main text.

For $\Phi_0 = 0$ V, the angle distributions obtained for the left and right hand side electrodes agree within the numerical accuracy (blue solid and dashed lines, Fig. S3), reflecting the

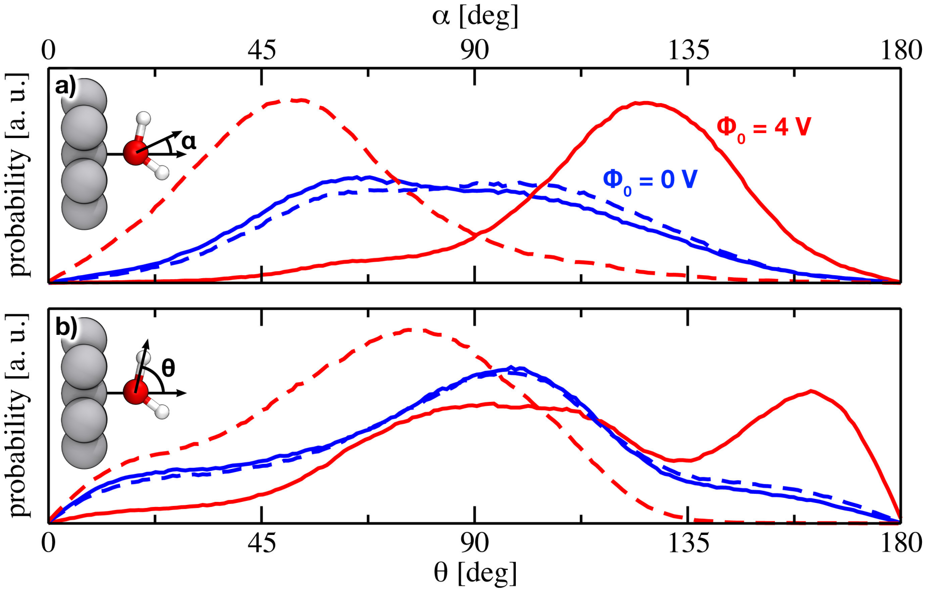

Figure S3: Probability distributions of **a)** the angle α enclosed between the surface normal and the water bisector and **b)** the angle θ enclosed between the surface normal and the water OH-bond. Solid and dashed lines indicate distributions computed for negatively and positively charged surfaces, respectively.

symmetry of our computational setup. Both the α and θ distributions are centered around 90°, indicating that for our hydrophobic electrodes interfacial water adopts largely planar configurations on average, where the molecular planes are parallel to the electrode surfaces.

At an applied voltage of $\Phi_0 = 4$ V, we observe a field-induced reorientation of the interfacial water molecules. On the negatively charged left hand electrode (solid red lines, Fig. S3) the interfacial water layer features a clear net dipole moment. The maximum of the probability distribution for the angle α between the water bisector and the surface normal is located at 126°. In agreement with recent findings by Li *et al.*[S8] for Au(111) surfaces, the OH-bond angle distribution becomes bimodal: one OH-bond remains in-plane (maximum at 96°), whereas the other OH-bond is now pointing towards the electrode surface (maximum at 162°) in an H-up configuration. On the positively charged right hand electrode (dashed red lines, Fig. S3), in contrast, such a bimodal distribution is absent since here the oxygen atoms of the water molecules are oriented towards the electrode surface.

We note that Li *et al.*[S8] used explicit counter ions to induce a surface charge. The inter-

facial water structure is sampled hence for surface charges that amount to an integer number of electrons and, by extension, for potentials that correspond to those integer charges. Moreover, the presence of the ions themselves may further modify the interfacial water structure. The thermopotentiostat approach introduced here, in contrast, allows us to perform simulations under potential control for arbitrary continuous potentials with and without ions present at the interface.

References

(S1) Deissenbeck, F.; Freysoldt, C.; Todorova, M.; Neugebauer, J.; Wippermann, S. Dielectric Properties of Nanoconfined Water: A Canonical Thermopotentiostat Approach. *Phys. Rev. Lett.* **2021**, *126*, 136803.

(S2) Gardiner, C. *Stochastic Methods*; Springer: Berlin, 2009.

(S3) Perdew, J. P.; Burke, K.; Ernzerhof, M. Generalized Gradient Approximation Made Simple. *Phys. Rev. Lett.* **1996**, *77*, 3865.

(S4) Blöchl, P. E. Projector augmented-wave method. *Phys. Rev. B* **1994**, *50*, 17953.

(S5) Kresse, G.; Hafner, J. Ab initio molecular dynamics for liquid metals. *Phys. Rev. B* **1993**, *47*, 558(R).

(S6) Kresse, G.; Furthmüller, J. Efficient iterative schemes for ab initio total-energy calculations using a plane-wave basis set. *Phys. Rev. B* **1996**, *54*, 11169.

(S7) Surendralal, S.; Todorova, M.; Finnis, M. W.; Neugebauer, J. First-Principles Approach to Model Electrochemical Reactions: Understanding the Fundamental Mechanisms behind Mg Corrosion. *Phys. Rev. Lett.* **2018**, *120*, 246801.

(S8) Li, C.-Y.; Le, J.-B.; Wang, Y.-H.; Chen, S.; Yang, Z.-L.; Li, J.-F.; Cheng, J.; Tian, Z.-

Q. In situ probing electrified interfacial water structures at atomically flat surfaces. *Nature Materials* **2019**, *18*, 697–701.
